# Automated Quantification of Pneumothorax in CT

**DOI:** 10.1155/2012/736320

**Published:** 2012-10-03

**Authors:** Synho Do, Kristen Salvaggio, Supriya Gupta, Mannudeep Kalra, Nabeel U. Ali, Homer Pien

**Affiliations:** ^1^Department of Radiology, Massachusetts General Hospital, 25 New Chardon Street, Suite 400B, Boston, MA 02114, USA; ^2^Albany Medical College, Albany, NY 12208, USA

## Abstract

An automated, computer-aided diagnosis (CAD) algorithm for the quantification of pneumothoraces from Multidetector Computed Tomography (MDCT) images has been developed. Algorithm performance was evaluated through comparison to manual segmentation by expert radiologists. A combination of two-dimensional and three-dimensional processing techniques was incorporated to reduce required processing time by two-thirds (as compared to similar techniques). Volumetric measurements on relative pneumothorax size were obtained and the overall performance of the automated method shows an average error of just below 1%.

## 1. Introduction

Pneumothorax is defined as the accumulation of air or gas in the space between the lung and the chest wall. It is a potentially life-threatening occurrence which frequently results from traumatic injuries. Although the exact percentage is in dispute, research indicates that pneumothoraces occur in approximately 20–50% of all chest injury cases [1–3]. Research has shown that in the absence of other significant injuries, or the need for intermittent positive-pressure ventilation, a majority of pneumothorax may be treated conservatively (observation, oxygen treatment, simple manual aspiration) without exposing the patient to the risks of intervention [4]. A primary factor in making the decision to conservatively treat a pneumothorax is the size of the air collection relative to the entire pleural region of the patient. Generally, pneumothorax occupying less than 20% of the hemithorax is said to be “small” and may be treated by observation alone [1, 5–8].

Multiple methods have been explored for estimating the size of pneumothorax utilizing chest radiography; however, these methods are merely estimation tools. Kircher and Swartzel [9] draws rectangles from reference points to demarcate the outlines of the hemithorax and lung and subtracts the respective areas to find percent pneumothorax. Another method proposed by Rhea et al. [10] predicts pneumothorax size by correlating average interpleural distance with radiographic thoracic gas volume measurements. Another study [11] proposed that the change in volume of the lung is equal to the cube of the change in its linear dimensions as visualized in radiographs. Determination of the size of a pneumothorax from two-dimensional radiographic images results in a large variance among users, and prescribed guidelines (such as interpleural distance) for measurement are prone to underestimating the true size of the pneumothorax [4, 7]. Further, patients who sustain trauma may have subtle pneumothorax that goes undetected on a chest radiograph due to restrictions in mobility of the patient for imaging purposes [12].

**Figure 1:**
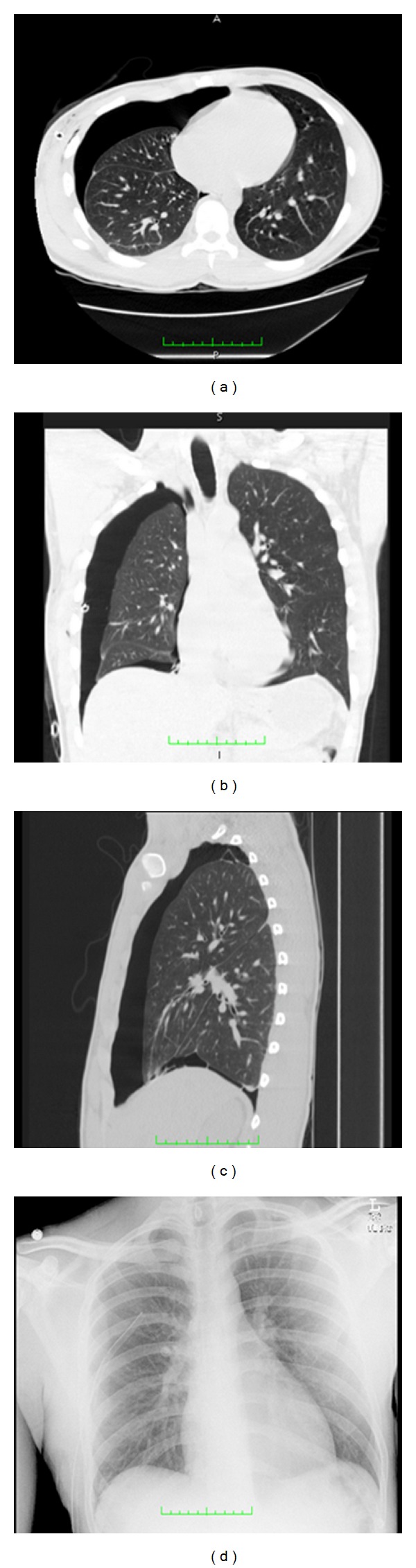
Transverse (a), coronal (b), and sagital (c) CT images of a patient with a moderate to large pneumothorax, and chest X-ray (d) of the same patient taken two hours prior to the time the CT images were acquired. CT images provide significantly better detail for detection and quantification of pneumothorax size.

Suspected pneumothorax may be screened with chest radiography; however, computed tomography (CT) provides several advantages. Occult pneumothorax exists in up to 50% of traumatic pneumothorax cases, which is undetectable on a chest X-ray. For this reason, it has long been recommended that CT of the chest should be performed in cases of suspected pneumothorax [1, 3, 13–15]. CT also provides physicians with the ability to more accurately quantify the size of the pneumothorax, assisting in treatment decisions (Figure 1). The drive towards conservative treatment of patients with smaller-sized pneumothorax presents the need for accurate quantification of pneumothorax size from CT datasets. Automated methods for generating such measurements in near real-time could help streamline patient care decisions, but to date little work has been done in this arena. We are aware of only one other study describing use of a computer-aided diagnosis (CAD) algorithm for detection and quantification of traumatic pneumothorax from CT datasets [16]. A frequent limitation of automated segmentation methods is that most are both time and computationally intensive.

There exists a need for an automated algorithm that allows objective and rapid assessment of pneumothorax imaged by CT. Current methods of evaluating pneumothorax by two-dimensional radiography yield inaccurate results and large reader variability due to subjective analysis. Imaging by CT allows for a more accurate and objective analysis by acquiring quantitative (objective) volumetric information. Automation further facilitates the objectivity of the analysis. The method would facilitate the conservative treatment of patients with smaller size pneumothorax. Current CAD methods are highly complex and relatively computation-intensive. The purpose of our study is to develop and assess an automated, simplified, and rapid CAD method for obtaining volumetric information with an accuracy similar to that obtained by manual segmentation.

## 2. Materials and Methods

Our institutional review board approved the retrospective collection of patient data for this study with waiver of informed consent. The study was conducted in compliance with the Health Insurance Portability and Accountability Act (HIPAA). 

### 2.1. Patients

This retrospective study included 8 patients with a mean age of 42 ± 20 years. There were 7 males and 1 female. All patients were scanned on a 16-section multidetector row CT scanner (GE Lightspeed Pro 16, GE Healthcare, Waukesha, WI, USA,) or a 64-section multidetector row CT scanner (GE Lightspeed VCT). Transverse sections were acquired with 120 kVp, automatic exposure control (Auto mA, GE Healthcare) using noise index of 25–35 and mA range of 75–440 mA, and 0.5 second gantry rotation speed. The detector configuration was 16 × 1.25 mm (for 16 section CT) or 64 × 0.625 mm (for 64 section CT). Transverse images were reconstructed to 2.5 section thickness at 2.5 mm increments using a detail soft-tissue reconstruction kernel. The datasets varied in number of sections from 116 to 159, with an average of 137 sections per dataset.

The patient datasets were collected via a keyword search through use for our institution's searchable radiology report and image repository. The datasets were selected to represent a range of pneumothorax sizes, spanning from normal to large, as defined in the radiology reports. Patients with underlying disease, coexisting hemothorax, or significant pleural effusion were excluded.

### 2.2. Automated Pneumothorax Segmentation

**Figure 2:**
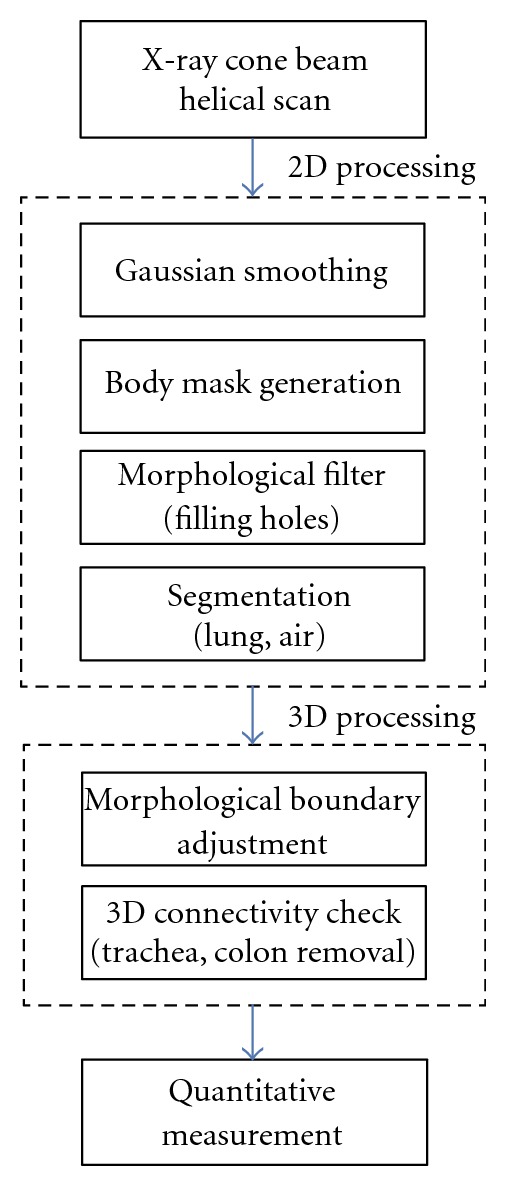
Flowchart displaying the order of operations included in the PTX CAD method. Regions within the pleural space containing air are identified and segmented during 2D processing; airways and other anatomies that are not constituting pneumothorax are removed during 3D processing.

An overview of our approach to segmentation of the lung and pneumothorax regions is shown in Figure 2. The approach consists of two distinct components: (1) 2D processing for rapid segmentation of the thoracic structures based on adaptive thresholding; and (2) 3D processing to correct for anatomical structures containing air outside of the pleural space. 

#### 2.2.1. 2*D* Processing with Adaptive Thresholding

Prior to segmentation, images (*f*(*x*, *y*)) were smoothed with a Gaussian filter, *g*(*x*, *y*) = 1/(2*πσ* ^2^)*e* ^−(*x*^2^ + *y*^2^)/2*σ*^2^^, where *σ* is the standard deviation of the Gaussian distribution. We used *σ* = 0.5 with a filter size of 5 × 5 to reduce noise and moderate textural variations within the images to better prepare for thresholding operations. Following Guassian smoothing, a fixed thresholding operation, Th_*M*_ = −500 Hounsfield Units (HU), was applied to generate a body mask to exclude all surrounding air outside of the patient's thorax. In applying this first thresholding operation, lung parenchyma and air within the thorax would also be excluded due to their lower intensity values, so a morphological reconstruction filter was applied to preserve these regions [17].

For morphological dilation and erosion, the mask may be regarded as a discrete Euclidian image, *A*(*m*, *n*) ∈ *Z* ^2^, with a network of points evenly distributed on a square grid. The pixels included in the image may only take on a value of 0 or 1. To fill holes within the body mask, the complement of the mask was computed, and a structuring element was propagated throughout the mask image, first by dilation and subsequently by erosion. Dilation by a disk structuring element corresponds to isotropic swelling or expansion to a binary mask. For an image, *A*, and a structuring element, *B*, in *Z* ^2^. The dilation of *A* by *B* is denoted by *A*  ⊕  *B* and is defined by:

(1) A⊕B={c∈Z2 ∣ c=a+b  for some  a∈A, b∈B}.

Dilation is commutative: *A* ⊕ *B* = *B* ⊕ *A* and associative: (*A*  ⊕  *B*) ⊕ *C* = *A* ⊕ (*B*  ⊕  *C*). The morphological dual of dilation is erosion, and the erosion of *A* by *B* is denoted by

(2) A⊖B={c∈Z2 ∣ c+b∈A  for  every  b∈B}.

When the reflection of *B* is denoted by $\overset{ˇ}{B}$ and is defined by

(3) Bˇ={c ∣ for  some  b∈B,c=−b},

the erosion and dilation duality can be defined by

(4) (A⊖B)c=Ac⊕Bˇ,

where *A* ^*c*^ = {*c* ∈ *Z* ^2^ | *c* ∉ *A*}. Also, with respect to structuring element decomposition, a chain rule for erosion holds when the structuring element is decomposable through dilation:

(5) A⊖(B⊕C)=(A⊖B)⊖C.

This relation is important because it permits a large erosion to be computed more efficiently by two or more successive smaller erosions [18]. We used a 2 × 2 disk structuring element for successive dilation and erosion in our application. Obtaining a complement of the result of the propagation forms the final mask.

For the segmentation of lung tissue and air, the analysis of the masked images' resulting histogram was performed to determine the two thresholding values in use representing air (Th_*A*_) and lung parenchyma (Th_*L*_) and an adaptive thresholding function was applied. The mean values for Th_*A*_ and Th_*L*_ were determined to be −870 ± 10 HU and −200 ± 15 HU, respectively.

#### 2.2.2. 3*D* Connectivity Constraint

Within the thorax, there are numerous regions which may contain air that would be included in the result of the adaptive thresholding operation, including the trachea, bronchi, and bowel. To remove these regions, morphological analysis on identified air components was performed. The centroid of each air component was calculated from the 2D images and its connectivity checked in 3D across neighboring slices. 

In a 3D data set, a connected component, *𝒜*, is a pile of 2D masks *A*_*l*_, where *l* = 1, 2,…, *L*, and *J* is the marker of the image. The reconstruction *Υ*_*𝒜*_(*J*) of a 3D mask *𝒜* from marker *J* is the union of the connected components of *𝒜*, which contain at least a pixel of *J*:

(6) Υ𝒜(J)=⋃J∩Ak≠∅Ak.

To be included as a pneumothorax, the air components identified during 2D processing must have finite boundaries within the pleural cavity; if there is continuity with air spaces outside of the chest cavity, such as exist with the bronchi or bowel, the air component is excluded.

#### 2.2.3. Volumetric Measurement

The volume of regions designated as air and lung tissue from the automated segmentation method is calculated (*V*_air_ and *V*_lung_) based on the pixel spacing values for each dataset. The resulting relative pneumothorax volume, *V*_ptx_, is defined as *V*_air_/(*V*_air_ + *V*_lung_) for a given patient.

### 2.3. Manual Segmentation

To assess the accuracy of the volumetric measurements obtained via the automated method, all 8 datasets were manually segmented using customized software developed in-house with MATLAB (Mathworks, Natick, MA, USA). Using the draggable point-drawing feature, manual contours were drawn to outline the total pleural region and the pneumothorax regions of each slice of each dataset. The resulting segmentations were subsequently reviewed and refined by an experienced thoracic radiologist.

### 2.4. Statistical Analysis

The relative pneumothorax volumes acquired from the automated and manual techniques were recorded and analyzed in Excel 2007 (Microsoft, Redmond, WA, USA) and SAS 9.2 (SAS, Cary, NC, USA). Absolute and average errors between the two volumes were assessed. The automated volumes were plotted against the manually obtained volumes and linear analysis was conducted. In addition, two statistical tests were used to evaluate the relationship between the obtained pneumothorax volumes. The paired *t*-test was used to assess whether the segmentation algorithms were statistically different across the samples examined, with the null hypothesis that the two distributions are the same; the Pearson moment product correlation coefficient was used to evaluate the null hypothesis that there was no correlation between the two methods. Execution times for the automated and manual methods were also recorded and averaged.

## 3. Results

**Figure 3:**
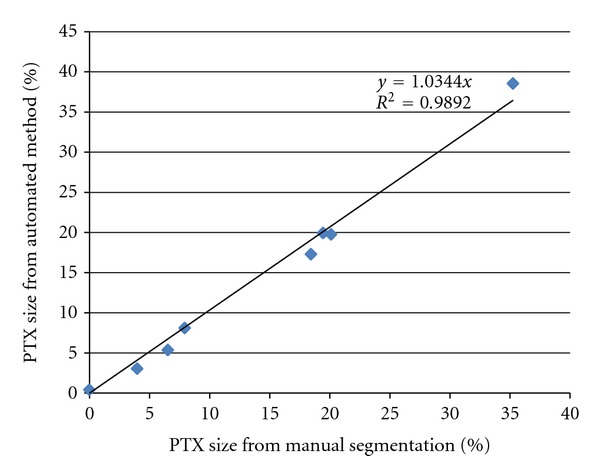
Plot displaying the measured size of PTX as a percentage of the total pleural space as compared between manual and automated methods. Trendline shows coefficient of determination at 0.989.

A comparison between the pneumothorax sizes obtained from the automated method and by manual segmentation showed strong correlation. The relative volumes obtained by the automated method were plotted against those obtained manually, and a linear trendline was applied (Figure 3). The absolute error between the automated and manual methods was calculated for each sample dataset and averaged to be 1% (Table 1). The paired *t*-test showed that the null hypothesis cannot be rejected, with *t* = 0.16 and *P* = 0.8771. The Pearson correlation was found to be 0.996, and the null hypothesis can be rejected at the 99.9% significance level. For further comparison between these methods and the original reporting of pneumothorax size, the qualitative size as indicated in the radiology report for each dataset was also tabulated alongside the manually obtained volume (Table 2).

**Figure 4:**
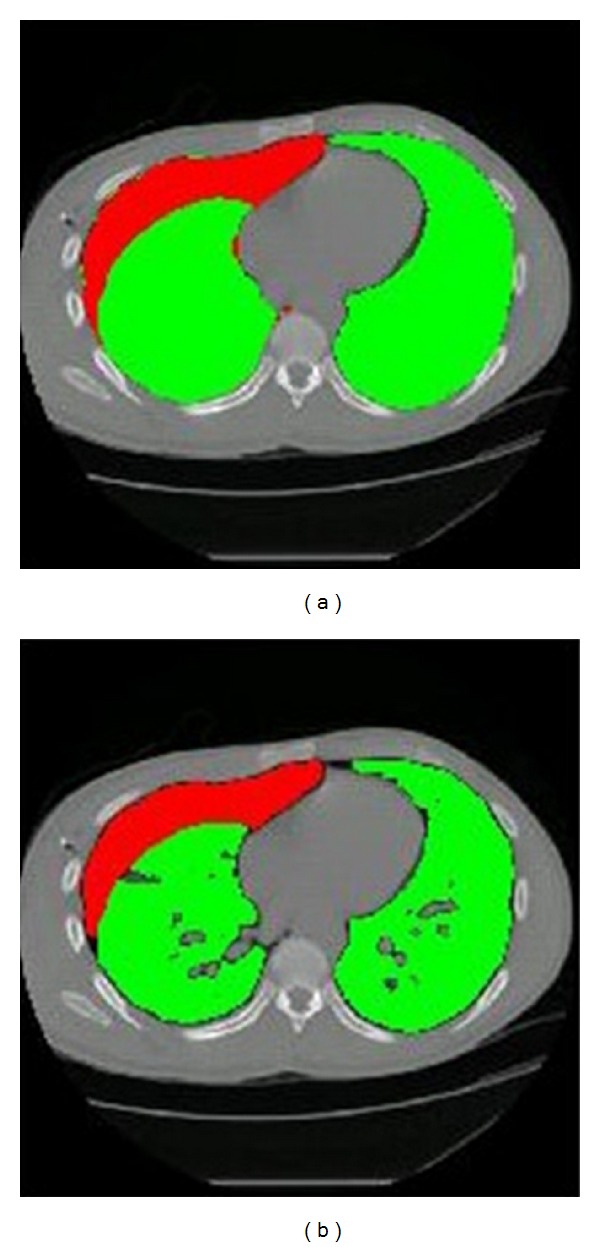
Segmented lung and air regions are displayed as green and red, respectively, for a single CT slice. (a) displays the segmentation result of the manual contours. (b) displays the segmentation result of the automated method after the 2D processing step, prior to 3D connectivity checks.

A visual display of a single section from a sample dataset shows the difference between manual segmentation and the first component of the automated method, 2D processing (Figure 4). The automated method is more sensitive to variations in tissue intensities within the lungs, as expected. Smaller airways and vessels, which were included as part of the lung during manual segmentation, were excluded during the first phase of the automated method. 

**Figure 5:**
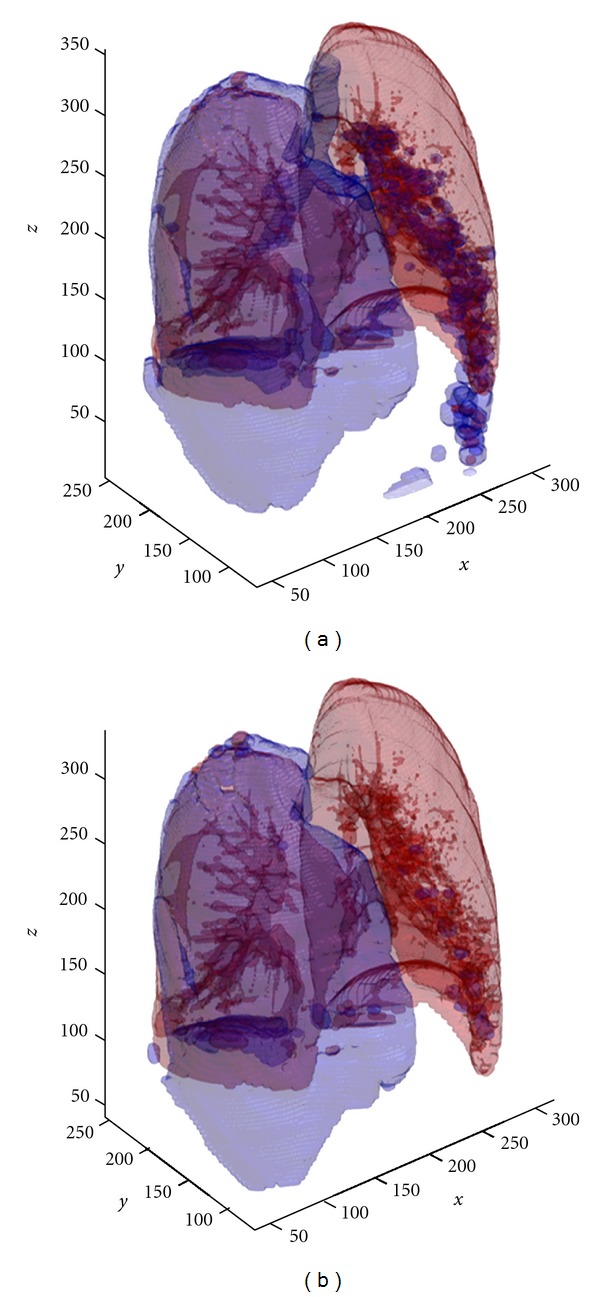
Segmented lung and air regions are displayed as red and blue, respectively. These images illustrate a large pneumothorax, as shown by the blue “air” region encompassing the right lung. (a) displays the result of the adaptive thresholding segmentation. (b) displays the result after applying the 3D connectivity constraints for the same case.

A second display of the same dataset before and after the second component of the automated method, application of 3D connectivity constraints, shows that the airways, including the bronchi, leading to the lungs have been largely removed, as well as parts of the bowel containing air (Figure 5).

The average execution time required by the automated method was 50.6 seconds per case using compiled MATLAB code running on a quad-core desktop PC. Manual segmentation through the use of customized MATLAB software required approximately 4.5 hours per dataset.

## 4. Discussion

Results show that the automated method produced measurements on the relative size of a pneumothorax that are comparable to those obtained by manual segmentation. Collectively, the statistical tests produced highly significant correlations between the two techniques. Furthermore, for an averaged sized dataset, the automated method was able to complete the calculations in less than one minute; this compares favorably against the approximately three minutes of processing cited by another study on CAD pneumothorax for similar datasets [16]. As noted above, these results were obtained using compiled MATLAB code on a quad-core desktop PC; it is anticipated that considerable acceleration could be achieved by further optimization, conversion to C/C++, and use of more specialized processing hardware to obtain similar results in no more than a few seconds. 

The algorithm differs significantly from other proposed methods in its relative simplicity. The other previously mentioned approach consisted of five distinct steps, involving multiple thresholding functions, including (1) a first pass of thresholding and region growing to segment the pleural space, with 3D morphological dilation to create a continuous region; (2) a second pass of thresholding for identification of pneumothorax candidates, with 3D morphological erosion to remove voxels included due to noise; (3) a third pass of dynamic thresholding to segment the suspected pneumothorax locations; (4) removal of the bronchus by location and boundary feature analysis; lastly, (5) removal of false positive detections by a radiologist prior to volumetric calculations [16]. In contrast, we applied a two-tiered algorithm involving fewer and simpler thresholding functions followed by a final 3D connectivity check to remove anatomical structures which may erroneously be included in the segmentation. This simplified approach has reduced average processing times by two-thirds without resulting in a loss of accuracy in quantification. Although absolute volume, instead of relative volume, was the value measured in the former study, the more elaborate algorithm achieved a correlation of 0.999 between automated and manual segmentation with a mean difference in volumes of approximately 7%. In comparison, our simplified method achieved a correlation of 0.996 and a mean difference in volumes of just under 1%. A thorough comparison between the two methods would require a larger sample size than was available for this study; however, these initial results show that a high level of accuracy may be acquired in a significantly reduced amount of time.

We note that in this study the “normal” patient was found to have a trace pneumothorax of size 0.38%. This error stems from the presence of the few voxels of air corresponding to normal airways that were not successfully eliminated during processing. While from a therapeutic perspective very small pneumothorax will not receive further intervention (merely monitoring), the question of how a clinician should deal with pneumothoraces found to be <0.5% in size by CAD algorithms still remains. Further, we did not assess the accuracy of the algorithm for quantifying pneumothorax in the presence of pleural effusion, hemothorax, or lung disease, such as emphysema. However, a similar limitation, that is, the exclusion of patients with these conditions, was noted in prior studies as well [16].

An interesting observation from the results of this study exemplified the wide discrepancy in the reporting of pneumothorax size among physicians, even among a limited sample size. Two out of the eight datasets corresponded to radiological reports which indicated that the patient had a “large” pneumothorax (cases 4 and 6); however, these same two datasets corresponded to manually obtained relative volumes of 18% and 35%. Two datasets characterized as “moderate,” each with a relative size of approximately 20% (cases 3 and 8), were actually greater in volume than one of the “large” pneumothorax. This observation illustrates the inexact nature of conventional (i.e., subjective) pneumothorax size assessment and presents the need for a more precise method of quantification. Although we did not assess the reason for discrepant reporting, it may be attributable to difficulty in visually or subjectively quantifying the extent of pneumothorax in a complex chest CT.

Obtaining results quickly is an important factor in determining the usefulness of an automated tool, particularly if applied in a trauma setting. Overall, our proposed automated pneumothorax analysis algorithm was found to be highly accurate relative to the ground truth of manual segmentation, while incurring minimal processing time. Further optimization to decrease computation time, and incorporation of methods to deal with complicating factors such as hemothoraces, would expand the utility of the algorithm.

## Figures and Tables

**Table 1:** Pneumothorax (PTX) size by method. The percentage size of the pneumothorax for each case is reported, as obtained from the automated method and by manual segmentation. The absolute error between the two methods is shown for each case and averages to be 0.99%.

	Case*1*	Case *2*	Case *3*	Case *4*	Case *5*	Case *6*	Case *7*	Case *8*
Auto PTX % size	8.10%	0.38%	19.73%	17.29%	5.34%	38.54%	3.04%	19.92%
Manual PTX % size	7.93%	0.00%	20.11%	18.44%	6.52%	35.26%	3.97%	19.45%
Absolute error	0.18%	0.38%	0.37%	1.16%	1.18%	3.27%	0.92%	0.46%

Average error	0.99%							

**Table 2:** Reported pneumothorax (PTX) Size. The qualitative size of each pneumothorax, as indicated on the radiological reports, is shown alongside the quantitative size of the pneumothorax obtained from manual segmentation.

	Case *1*	Case *2*	Case *3*	Case *4*	Case *5*	Case *6*	Case *7*	Case *8*
Reported size	Small to moderate	Normal	Moderate	Large	Small	Large	Small to moderate	Moderate
Manual PTX % size	7.93%	0.00%	20.11%	18.44%	6.52%	35.26%	3.97%	19.45%
